# Influence of single-nucleotide polymorphisms in *TLR3* (rs3775291) and *TLR9* (rs352139) on the risk of CMV infection in kidney transplant recipients

**DOI:** 10.3389/fimmu.2022.929995

**Published:** 2022-07-29

**Authors:** Natalia Redondo, Isabel Rodríguez-Goncer, Patricia Parra, Tamara Ruiz-Merlo, Francisco López-Medrano, Esther González, Natalia Polanco, Hernando Trujillo, Ana Hernández, Rafael San Juan, Amado Andrés, José María Aguado, Mario Fernández-Ruiz

**Affiliations:** ^1^ Unit of Infectious Diseases, Hospital Universitario “12 de Octubre”, Instituto de Investigación Sanitaria Hospital “12 de Octubre” (imas12), Madrid, Spain; ^2^ Centro de Investigación Biomédica en Red de Enfermedades Infecciosas (CIBERINFEC), Madrid, Spain; ^3^ Department of Medicine, School of Medicine, Universidad Complutense, Madrid, Spain; ^4^ Department of Nephrology, Hospital Universitario “12 de Octubre”, Instituto de Investigación Sanitaria Hospital “12 de Octubre” (imas12), Madrid, Spain

**Keywords:** cytomegalovirus, kidney transplantation, single-nucleotide polymorphism (SNP), toll-like receptor 3 (TLR3), toll-like receptor 9 (TLR9)

## Abstract

Risk stratification for cytomegalovirus (CMV) infection after kidney transplantation (KT) remains to be determined. Since endosomal toll-like receptors (TLRs) are involved in viral sensing, we investigated the impact of common single-nucleotide polymorphisms (SNPs) located within *TLR3* and *TLR9* genes on the occurrence of overall and high-level (≥1,000 IU/ml) CMV infection in a cohort of 197 KT recipients. Homozygous carriers of the minor allele of *TLR3* (rs3775291) had higher infection-free survival compared with reference allele carriers (60.0% for TT versus 42.3% for CC/CT genotypes; *P*-value = 0.050). Decreased infection-free survival was observed with the minor allele of *TLR9* (rs352139) (38.2% for TC/CC versus 59.3% for TT genotypes; *P*-value = 0.004). After multivariable adjustment, the recessive protective effect of the *TLR3* (rs3775291) TT genotype was confirmed (adjusted hazard ratio [aHR]: 0.327; 95% CI: 0.167–0.642; *P*-value = 0.001), as was the dominant risk-conferring effect of *TLR9* (rs352139) TC/CC genotypes (aHR: 1.865; 95% CI: 1.170–2.972; *P*-value = 0.009). Carriers of the *TLR9* (rs352139) TC/CC genotypes showed lower CMV-specific interferon-γ-producing CD4+ T-cell counts measured by intracellular cytokine staining compared with the TT genotype (median of 0.2 versus 0.7 cells/μl; *P*-value = 0.003). In conclusion, *TLR3/TLR9* genotyping may inform CMV infection risk after KT.

## Introduction

Compared to non-transplant subjects, solid organ transplant (SOT) recipients are at an increased risk of infectious complications due to the sustained effect of immunosuppressive therapies (1, 2). Current regimens are mainly aimed at abolishing adaptive cell-mediated responses, rendering the innate immune system a relevant player in the host defense against opportunistic pathogens (2). Cytomegalovirus (CMV) constitutes a major cause of morbidity and mortality in the SOT population (3). Following primary infection and similar to other members of the *Herpesviridae* family, CMV establishes life-long latency, which is usually controlled by the immune system. In certain circumstances—such as iatrogenic immunosuppression or biological stress—CMV may reactivate and cause clinically evident disease (4).

Despite advances in the understanding of the role of CMV-specific immunity over recent years, the choice of the optimal prevention strategy against CMV after SOT remains essentially informed by two factors only: donor and recipient (D/R) serostatus and previous use of T-cell-depleting agents (5, 6). The burden of post-transplant CMV infection and disease observed in large contemporary cohorts (7, 8), however, suggests that there is still room for improvement in risk stratification strategies (9). Increasing evidence supports the impact of single-nucleotide polymorphisms (SNPs) in genes that orchestrate innate and adaptive responses on the risk of CMV infection and disease (3, 10). Most of these studies have focused on pattern recognition receptors (PRRs). The major family of PRRs are toll-like receptors (TLRs), which may be classified according to their cellular location and ligand recognition specificity. Membrane-bound TLRs (such as TLR2, TLR4, or TLR5) primarily recognize pathogen-associated molecular patterns (PAMPs) of bacterial origin, whereas TLRs located within endosomal compartments (TLR3, TLR7, TLR8, and TLR9) predominantly bind viral nucleic acids (11, 12).

TLR3 recognizes extracellular double-stranded RNA (dsRNA), a by-product generated during replication and transcription of most viruses (13). It has been well-established the effect of genetic defects in the TLR3 pathway on the development of central nervous system (CNS) infection due to herpes simplex virus (HSV) and varicella-zoster virus (VZV) among otherwise immunocompetent individuals (14–16). Comparable data for the susceptibility to CMV in SOT recipients is still lacking. On the other hand, TLR9 senses and is activated by unmethylated cytosine-phosphate-guanine (CpG) dinucleotides. Various studies have reported that newborns carrying certain SNPs in the *TLR9* gene (e.g., rs187084, rs352140) are at an increased risk of congenital CMV infection (17–19). By using a large multicenter cohort of seropositive kidney transplant (KT) recipients (OPERA Study), we first reported that the presence of the TT genotype in the *TLR9* (rs5743836) SNPs provided protection against CMV viremia (20). However, mo further studies have investigated whether genetic variations in *TLR9* are associated with the risk of developing post-transplant CMV infection or disease (10).

Given this paucity of data and the critical involvement of endosomal TLR-mediated signaling pathways in viral sensing, we aimed to investigate the influence of common SNPs in the *TLR3* (rs3775291) and *TLR9* (rs352139, rs5743836) genes on the incidence of CMV events in a cohort of KT recipients. The rs3775291 SNP is located within exon 4 of the *TLR3* gene and implies a variation of 1234C>T, which changes a conserved leucine by a phenylalanine at position 412 (21). In relation to SNPs located in *TLR9*, rs352139 is located in intron 1 of exon 2 (1174T>C), whereas rs5743836 SNP is situated in the promoter region of the gene at position −1237A>G. In both cases, the polymorphism impacts *TLR9* expression (22, 23). We also attempted to correlate the presence of genetic determinants of CMV infection with functional markers of CMV-specific T-cell-mediated immunity (CMV-CMI).

## Patients and methods

### Study population and setting

This observational study was based on a prospectively maintained database that included all consecutive adult patients undergoing KT at the University Hospital “12 de Octubre” between November 2014 and December 2016. Double organ recipients and those suffering from graft loss within the first post-transplant week were excluded. For this study, we also excluded patients at low risk for CMV infection (D−/R−). Participants were enrolled at the time of transplantation and followed up for at least 12 months, unless graft loss (i.e., retransplantation or return to dialysis) or death occurred earlier. Scheduled follow-up visits were carried out at baseline, every 2 weeks during the first 3 months, and monthly thereafter, as well as whenever clinically indicated. Pre-transplant, peri-operative, and post-transplant variables were prospectively recorded by means of a standardized case report form, and pseudo-anonymized data were entered into a secure REDCap database. Descriptions of immunosuppression and prophylaxis regimens are detailed in **Supplementary Methods**. The study was performed in accordance with the ethical standards outlined in the Declarations of Helsinki and Istanbul. All the patients provided informed consent and the local Clinical Research Ethics Committee approved the protocol (number 14/030). This study was prepared in accordance with the methodological recommendations drawn by the STREGA initiative.

### Study design and study definitions

The primary study outcomes were the occurrence of overall CMV infection and high-level CMV infection (defined by a viral load ≥1,000 IU/ml) during the first year after transplantation. This cut-off value of ≥1,000 IU/ml is the threshold usually applied in our institution to initiate preemptive antiviral therapy in KT recipients with asymptomatic CMV viremia. The development of CMV disease during that period was considered a secondary outcome. The diagnosis of CMV infection required polymerase chain reaction (PCR)-confirmed CMV replication regardless of the presence of attributable symptoms. The CMV viral load was quantified as previously described (24). Briefly, 200 μl of whole blood was used for DNA extraction using a NucliSENS^®^ easyMag^®^ instrument (bioMérieux Diagnostics, Marcy l’Etoile, France), according to the instructions of the manufacturer. A commercial real-time PCR assay (RealStar^®^ CMV PCR kit 1.0, Altona Diagnostics GmbH, Hamburg, Germany) was used for viral DNA quantification. CMV viremia was assessed per protocol fortnightly during the first 2 months and then monthly thereafter during the first year. Additionally, real-time PCR was performed whenever CMV disease was suspected. CMV disease included viral syndrome or end-organ disease and required the documentation of CMV viremia along with attributable symptoms (25), as detailed in **Supplementary Methods**.

### SNP genotyping

Whole blood specimens were collected in EDTA tubes at patient inclusion and stored at −80°C until analysis. DNA was extracted with the KingFisher™ Duo Prime system (Thermo Fisher Scientific, Waltham, MA) using the MagMax™ DNA Multi-Sample Ultra 2.0 kit, following the instructions of the manufacturer. *TLR3* (rs3775291) and *TLR9* (rs5743836, rs352139) genotyping was performed by TaqMan technology (Thermo Fisher Scientific) in a QuantStudio 3 system (Applied Biosystems, Foster City, CA). Sequences of TaqMan probes were as follows: ACTTGCTCATTCTCCCTTACACATA**[T/C]**TCAACCTAACCAAGAATAAAATCTC for *TLR3* (rs3775291), TGGGATGTGCTGTTCCCTCTGCCTG**[A/G]**AAACTCCCCCAAGTCTCATATGACC for *TLR9* (rs5743836) and TGTGTGAGTGGCCGGCCCCCAGCTC**[C/T]**ACCTCCACCCACTCCACTTCATGGG for *TLR9* (rs352139). SNP and allele (genotype) calling was carried out by a standard end-point analysis with the aid of commercial genotype-calling software (TaqMan™ Genotyper Software v1.0.1) and the QuantStudio Design and Analysis Software v1.5.1 (both from Applied Biosystems).

### Assessment of CMV-CMI

The magnitude and functionality of the CMV-CMI were investigated by two different methods. We used a commercial enzyme-linked immunosorbent assay (ELISA)-based interferon (IFN)-γ release assay (QuantiFERON^®^-CMV [QTF-CMV], Qiagen GmbH, Hilden, Germany) according to the manufacturer’s instructions and as described elsewhere (26). Additionally, we performed in a second subgroup the intracellular cytokine staining (ICS) method with flow cytometry to enumerate CMV pp65 and immediate-early (IE)-1-specific IFN-γ-producing CD8+ and CD4+ T cells, as previously described (27). Details on both techniques are available in **Supplementary Methods**.

### Statistical analysis

Quantitative data are shown as the mean ± standard deviation (SD) or the median with interquartile range (IQR). The qualitative variables are expressed as absolute and relative frequencies. The normality of the distributions was tested with the Kolgomorov–Smirnov test. The deviation from the Hardy–Weinberg equilibrium (HWE) for each SNP was evaluated by the χ^2^ test with one degree of freedom. Comparisons of the cumulative incidence of study outcomes (overall and high-level CMV infection and CMV disease) according to different genotypes for the SNPs investigated were performed by the χ^2^ test or the Fisher’s exact test, as appropriate. Additional pairwise comparisons were conducted between different SNP genotype groups, either individually or in combination. Survival probabilities were estimated by the Kaplan–Meier method with study outcomes as events, and differences between groups were compared using the log-rank test. Multivariable Cox regression models were constructed with study outcomes as the dependent variables and using the entered method for entering the explicative variables. Results were expressed as hazard ratios (HRs) and 95% confidence intervals (CIs). Models were adjusted by those variables with a *P*-value <0.05 at the univariate level. Multicollinearity was analyzed with the variance inflation factor, with values of <3 being considered acceptable. We also performed a haplotype analysis by creating a score based on the SNP genotypes found to exert an independent impact on the primary outcomes. All the significance tests were two-tailed and considered significant at a *P*-value <0.05. Statistical analysis was performed using SPSS v21 (Statistical Package for Social Sciences, Chicago, IL) and graphs were generated with Prism v6.0 (GraphPad Software Inc., La Jolla, CA).

## Results

### Study population and outcomes

Overall, we included 197 KT recipients. As detailed in **Table 1**, 111 patients (56.3%) received valganciclovir prophylaxis for a median of 103 days (IQR: 91–148). The median follow-up period was 1,122 days (IQR: 956–1,297), and all but six patients (97.1%) completed the one-year follow-up with a functioning graft. The only relevant modification in the immunosuppression regimen was the conversion to a mammalian target of rapamycin (mTOR) inhibitor (target trough level of 4–6 ng/ml), which was performed on an individual basis beyond the third month in 19 patients (9.6%) due to the occurrence of tacroIimus-related adverse effects, difficult-to-treat CMV or BK infection, or post-transplant malignancy. Ten patients (5.1%) died at a median interval of 917.5 days (IQR: 600.3–1,390) from transplantation, and eight patients (4.1%) experienced graft loss. One- and two-year survival rates were 97.9% and 96.6%, whereas death-censored graft survival was 98.5% and 96.5%, respectively.

**Table 1:** Demographics and clinical characteristics of the study cohort (n = 197).

Variable	
Age, years [mean ± SD]	55.1 ± 15.4
Gender (male) [n (%)]	141 (71.6)
Body mass index, kg/m^2^ [mean ± SD]^a^	26.0 ± 9.6
Ethnicity [n (%)]	
Caucasian	170 (86.3)
Hispanic	17 (8.6)
African	6 (3.0)
Asian	4 (2.0)
Current or prior smoking history [n (%)]	80 (40.6)
Pre-transplant chronic comorbidities [n (%)]	
Hypertension	169 (85.8)
Diabetes mellitus	57 (28.9)
Chronic lung disease	27 (13.7)
Coronary heart disease	21 (10.7)
Other chronic heart disease	35 (17.8)
Peripheral arterial disease	21 (10.7)
Cerebrovascular disease	16 (8.1)
Previous kidney transplantation [n (%)]	26 (13.2)
Underlying end-stage renal disease [n (%)]	
Diabetic nephropathy	34 (17.3)
Polycystic kidney disease	23 (11.7)
Glomerulonephritis	46 (23.4)
Nephroangiosclerosis	18 (9.1)
Congenital nephropathy	6 (3.0)
Reflux nephropathy	7 (3.6)
NSAID-associated nephropathy	3 (1.5)
Unknown	24 (12.2)
Other	26 (13.2)
CMV serostatus [n (%)]	
D+/R+	148 (75.1)
D+/R−	23 (11.7)
D−/R+	22 (11.2)
D not available/R+	4 (2.0)
Positive HCV serostatus [n (%)]^b^	15 (7.6)
Positive HIV serostatus [n (%)]^c^	2 (1.0)
Pre-transplant renal replacement therapy [n (%)]	175 (88.8)
Hemodialysis	131/175 (80.4)
Continuous ambulatory peritoneal dialysis	32/163 (19.6)
Time on dialysis, months [median (IQR)]	17.2 (8.9–36.0)
Age of donor, years [mean ± SD]	53.9 ± 15.5
Gender of donor (male) [n (%)]	105 (53.3)
Type of donor [n (%)]	
DBD donor	125 (63.5)
DCD donor	45 (22.8)
Living donor	26 (13.2)
Cold ischemia time, hours [median (IQR)]	18.0 (10.4–23.0)
Number of HLA mismatches [median (IQR)]	4 (3–5)
Induction therapy [n (%)]	
ATG	92 (46.7)
Basiliximab	79 (40.1)
None	26 (13.2)
Primary immunosuppression regimen [n (%)]	
Prednisone, tacrolimus and MMF/MPS	182 (92.4)
Prednisone, tacrolimus and azathioprine	8 (4.1)
Prednisone and tacrolimus	6 (3.0)
Prednisone, cyclosporine and MMF/MPS	1 (0.5)
Conversion to mTOR inhibitor during follow-up [n (%)]	19 (9.6)
Time to conversion, days [median (IQR)]	232 (118–321)
Anti-CMV prophylaxis [n (%)]	111 (56.3)
Duration of VGCV prophylaxis, days [median (IQR)]^d^	103 (91–148)
≤90 days [n (%)]	56 (28.4)
90–180 days [n (%)]	24 (12.2)
≥180 days [n (%)]	25 (12.7)
Post-transplant complications [n (%)]	
Delayed graft function	98 (49.7)
Reintervention within the first month	19 (9.6)
New-onset diabetes after transplantation	23 (11.7)
Renal artery stenosis	38 (19.3)
Acute graft rejection	23 (11.7)
More than on episode of rejection	5 (2.5)
Interval from transplantation to the first episode, days [median (IQR)]	142 (25.5–502.0)

ATG, antithymocyte globulin; CMV, cytomegalovirus; D, donor; DBD, donation after brain death; DCD, donation after circulatory death; HCV, hepatitis C virus; HIV, human immunodeficiency virus; HLA, human leukocyte antigen; IQR, interquartile range; MMF/MPS, mycophenolate mofetil/mycophenolate sodium; mTOR, mammalian target of rapamycin; NSAID, nonsteroidal anti-inflammatory drug; R, recipient; SD, standard deviation; VGCV, valganciclovir.

a Data on body mass index of the recipient was not available for 17 patients.

b Data on the HCV serostatus was not available for five patients.

c Data on the HIV serostatus was not available for two patients.

d Data on the duration of VGCV prophylaxis was not available for six patients.

By month 12, 109 patients had developed at least one episode of CMV infection, resulting in a one-year cumulative incidence of 55.3% (95% CI: 48.1–62.4). The median interval from the first episode of infection was 73 days (IQR: 38.5–149.5). The one-year cumulative incidence of high-level CMV infection was 36.5% (29.8–43.7). Regarding the secondary outcome, 22 patients experienced CMV disease, which comprised viral syndrome (18 patients [81.8%]), colitis (3 patients [13.6%]) and hepatitis (one patient [4.5%]), yielding a one-year cumulative incidence of 11.2% (95% CI: 7.1–16.4).

### Effect of *TLR3* (rs3775291) and *TLR9* (rs5743836, rs352139) SNPs on the risk of CMV infection

All the samples were successfully genotyped. The genotype frequency distribution of each SNP is detailed in **Table S1** of the Supporting Material. The *TLR9* (rs5743836) SNP significantly deviated from the HWE. First, we investigated whether the presence of specific alleles within these SNPs was correlated with the cumulative incidence of CMV infection in the study population. As observed in **Table 2**, carriers of the minor *TLR3* allele in the homozygous state (TT) had a lower 12-month incidence of CMV infection (40.0%) compared than homozygotes (CC) or heterozygotes (CT) for the reference allele (51.0% and 66.2%, respectively) (*P*-value = 0.036). A numerical difference was also observed for *TLR9* (rs5743836), where the alternative G allele both in heterozygous (AG) or homozygous states (GG) was apparently associated with CMV infection (cumulative incidence rates of 64.4% and 66.7%, respectively) compared to the AA genotype (51.7%), although the difference did not reach statistical significance *(P*-value = 0.256). On the other hand, the minor C allele of *TLR9* (rs352139) was also associated with an increased incidence of CMV infection, either in the heterozygous (TC) or homozygous state (CC) (56.8% and 66.7%, respectively) compared with homozygous patients (TT) for the reference allele (40.7%) *(P*-value = 0.017).

**Table 2:** One-year cumulative incidence of CMV infection according to the genotypic distribution of the candidate SNPs.

Gene (SNP database ID number)	Genotype	N	CMV infection by month 12 (n [%])		*P*-value
			No infection (n = 88)	Infection (n = 109)		
*TLR3* (rs3775291)	CC	98	48 (49.0)	50 (51.0)	0.036
	CT	74	25 (33.8)	49 (66.2)	
	TT	25	15 (60.0)	10 (40.0)	
*TLR9* (rs5743836)	AA	143	69 (48.3)	74 (51.7)	0.256
	AG	45	16 (35.6)	29 (64.4)	
	GG	9	3 (33.3)	6 (66.7)	
*TLR9* (rs352139)	TT	59	35 (59.3)	24 (40.7)	0.017
	TC	87	36 (41.4)	51 (56.8)	
	CC	51	17 (33.3)	34 (66.7)	

CMV, cytomegalovirus; ID, identification; SNP, single-nucleotide polymorphism; TLR, toll-like receptor.

Next, we evaluated the impact of minor alleles in rs3775291, rs5743836, and rs352139 on the susceptibility to CMV in dominant (homozygous or heterozygous states) and recessive models (homozygous state only). In the case of the *TLR3* (rs3775291) SNP, we found a trend toward a lower incidence of CMV infection among carriers of the T allele in the homozygous state (40.0% for TT versus 57.6% for CC/CT; *P*-value = 0.099), suggestive of a recessive protective effect. Regarding the *TLR9* gene, the presence of the minor allele in homozygous or heterozygous states was associated with the occurrence of CMV infection, both for rs5743836 (64.8% for AG/GG versus 51.7% for AA; *P-*value = 0.1) and rs352139 (61.6% for TC/CC versus 40.7% for TT; *P-*value = 0.007), indicative of a dominant risk conferring effect (**Table 3**).

**Table 3:** One-year cumulative incidence of CMV infection according to recessive and dominant models for the minor alleles of candidate SNPs.

Gene (SNP database ID number)	Model	Genotype	N	CMV infection by month 12 (n [%])		*P*-value
					No infection (n = 88)		Infection (n = 109)
*TLR3* (rs3775291)	Dominant	CC	98	48 (49.0)	50 (51.0)	0.226
		CT/TT	99	40 (40.4)	59 (59.6)
	Recessive	CC/CT	172	73 (42.4)	99 (57.6)	0.099
		TT	25	15 (60.0)	10 (40.0)
*TLR9* (rs5743836)	Dominant	AA	143	69 (48.3)	74 (51.7)	0.100
		AG/GG	54	19 (35.2)	35 (64.8)
	Recessive	AA/AG	188	85 (45.2)	103 (54.8)	0.484
		GG	9	3 (33.3)	6 (66.7)
*TLR9* (rs352139)	Dominant	TT	59	35 (59.3)	24 (40.7)	0.007
		TC/CC	138	53 (38.4)	85 (61.6)
	Recessive	TT/TC	146	71 (48.6)	75 (51.4)	0.059
		CC	51	17 (33.3)	34 (66.7)

CMV, cytomegalovirus; ID, identification; SNP, single-nucleotide polymorphism; TLR, toll-like receptor.

We also explored the effect of candidate SNPs on the development of high-level CMV infection during the first post-transplant year (**Table S2**). Consistent with the associations observed for CMV viremia at all levels, the incidence of this outcome was lower among carriers of the *TLR3* (rs3775291) TT genotype, although the difference did not reach statistical significance (20.0% [5/25] versus 39.0% [67/172]; *P*-value = 0.066). The opposite dominant effect was confirmed for both *TLR9* SNPs, with an increased incidence of high-level infection among homozygous or heterozygous carriers of the minor alleles of rs5743836 (48.1% [26/54] versus 32.2% [46/143]; *P*-value = 0.038) and rs352139 (41.3% [57/138] versus 25.4% [15/59]; *P*-value = 0.034).

### Impact of selected genotypes of candidate SNPs on CMV infection-free survival

**Figure 1:**
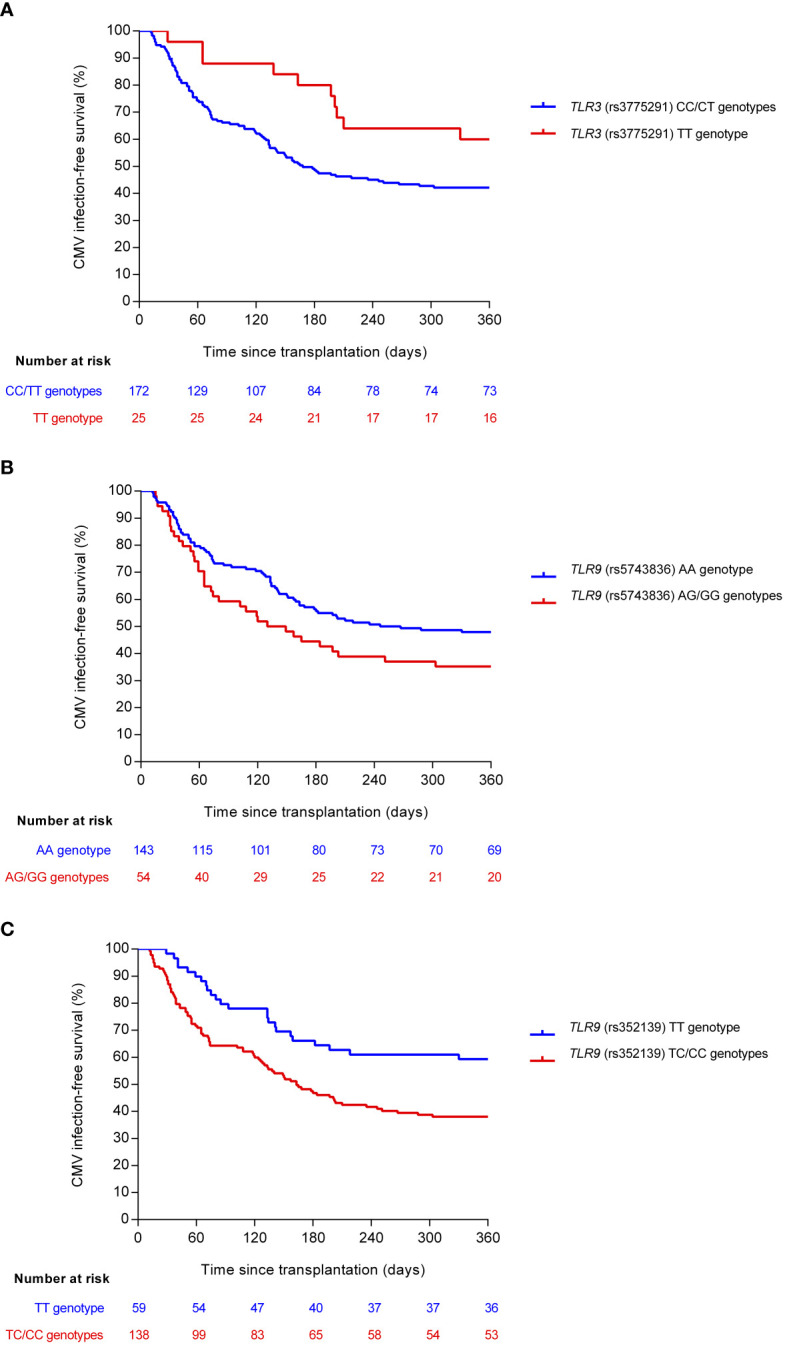
Comparison of CMV infection-free survival according to selected genotypes of candidate SNPs: **(A)** *TLR3* (rs3775291) (log-rank *P*-value = 0.050), **(B)** *TLR9* (rs5743836) (log-rank *P*-value = 0.063), and **(C)** *TLR9* (rs352139) (log-rank *P*-value = 0.004). Reference and minor alleles are shown as blue and red curves, respectively. CMV, cytomegalovirus; SNP, single-nucleotide polymorphism.

In view of the associations observed between candidate SNPs in the *TLR3* and *TLR9* genes and the one-year cumulative incidence of overall and high-level CMV infection, we plotted event free-survival curves according to the selected genotype combinations (**Figure 1**). Homozygous carriers of the minor T allele of *TLR3* (rs3775291) were more likely to remain free from CMV infection compared with carriers of the reference allele in homozygous or heterozygous states (CC/CT) (one-year survival rates: 60.0% versus 42.3%; log-rank test *P*-value = 0.050). In contrast, patients carrying the minor G allele (AG/GG) of *TLR9* (rs5743836) had lower CMV infection-free survival than homozygous carriers of the reference allele (AA) (one-year survival rates: 35.2% versus 48.1%; log-rank test *P*-value = 0.063). Similar findings were found for the minor C allele of *TLR9* (rs352139) (one-year survival rates: 38.2% versus 59.3% for TC/CC and TT genotypes, respectively; log-rank test *P*-value = 0.004).

Comparable results were obtained for high-level (≥1,000 IU/ml) CMV infection. Indeed, there was a non-significant trend toward an increased CMV infection-free survival among the homozygous carriers of the *TLR3* (rs3775291) protective T allele (log-rank test *P*-value = 0.066). On the other hand, the presence of the minor risk alleles within the *TLR9* SNPs—AG/GG for rs5743836 and TC/CC for rs352139—was associated with a decreased probability of remaining free from high-level infection during the first year (log-rank test *P*-values = 0.031 and 0.024, respectively) (**Figure S1**).

We subsequently tested by multivariable analysis the impact of these SNPs on study outcomes. To this end, Cox regression models were adjusted by clinical variables found to be associated at the univariable level with the occurrence of overall (**Table S3**) and high-level CMV infection (**Table S4**). After controlling for recipient and donor age, D+/R− CMV serostatus, use of antiviral prophylaxis, and graft function by month 1, the protective effect of the minor allele of the *TLR3* (rs3775291) SNP in the homozygous state (TT) was confirmed (adjusted HR [aHR]: 0.327; 95% CI: 0.167–0.642; *P*-value = 0.001). No independent effect was observed for *TLR9* (rs5743836) (aHR: 1.260; 95% CI: 0.836–1.899; *P*-value = 0.269), whereas the presence of the minor allele of *TLR9* (rs352139) in the dominant model (TC/CC) had a significant risk effect (aHR: 1.865; 95% CI: 1.170–2.972; *P*-value = 0.009) (**Table 4**). Regarding the high-level CMV infection, the *TLR9* (rs352139) SNP was found to act as an independent risk factor (aHR: 1.859; 95% CI: 1.039–3.328; *P*-value = 0.037) and the *TLR3* (rs3775291) SNP to play a protective role (aHR: 0.409; 95% CI: 0.162–1.036; *P*-value = 0.059) (**Table S5**).

**Table 4:** Multivariable Cox regression models assessing the impact of selected SNPs on the incidence of CMV infection during the first post-transplant year.

Genotype	aHR^a^	95% CI	*P-*value
TT genotype of *TLR3* (rs3775291) SNP (*vs*. CC/CT)	0.327	0.167–0.642	0.001
AG/GG genotype of *TLR9* (rs5743836) SNP (*vs*. AA)	1.260	0.836–1.899	0.269
TC/CC genotype of *TLR9* (rs352139) SNP (*vs*. TT)	1.865	1.170–2.972	0.009
Number of unfavorable genotypes	1.528^b^	1.212–1.927	<0.001

aHR, adjusted hazard ratio; CI, confidence interval; SNP, single-nucleotide polymorphism; TLR, toll-like receptor.

a Model adjusted for recipient and donor age, D+/R− CMV serostatus, receipt of valganciclovir prophylaxis and graft function at month 1. D+/R+ CMV serostatus and induction therapy with ATG were not included due to their high collinearity with D+/R− serostatus and the receipt of antiviral prophylaxis, respectively.

b HR per each one-genotype increment.

### Additive effect of risk genotypes

**Figure 2:**
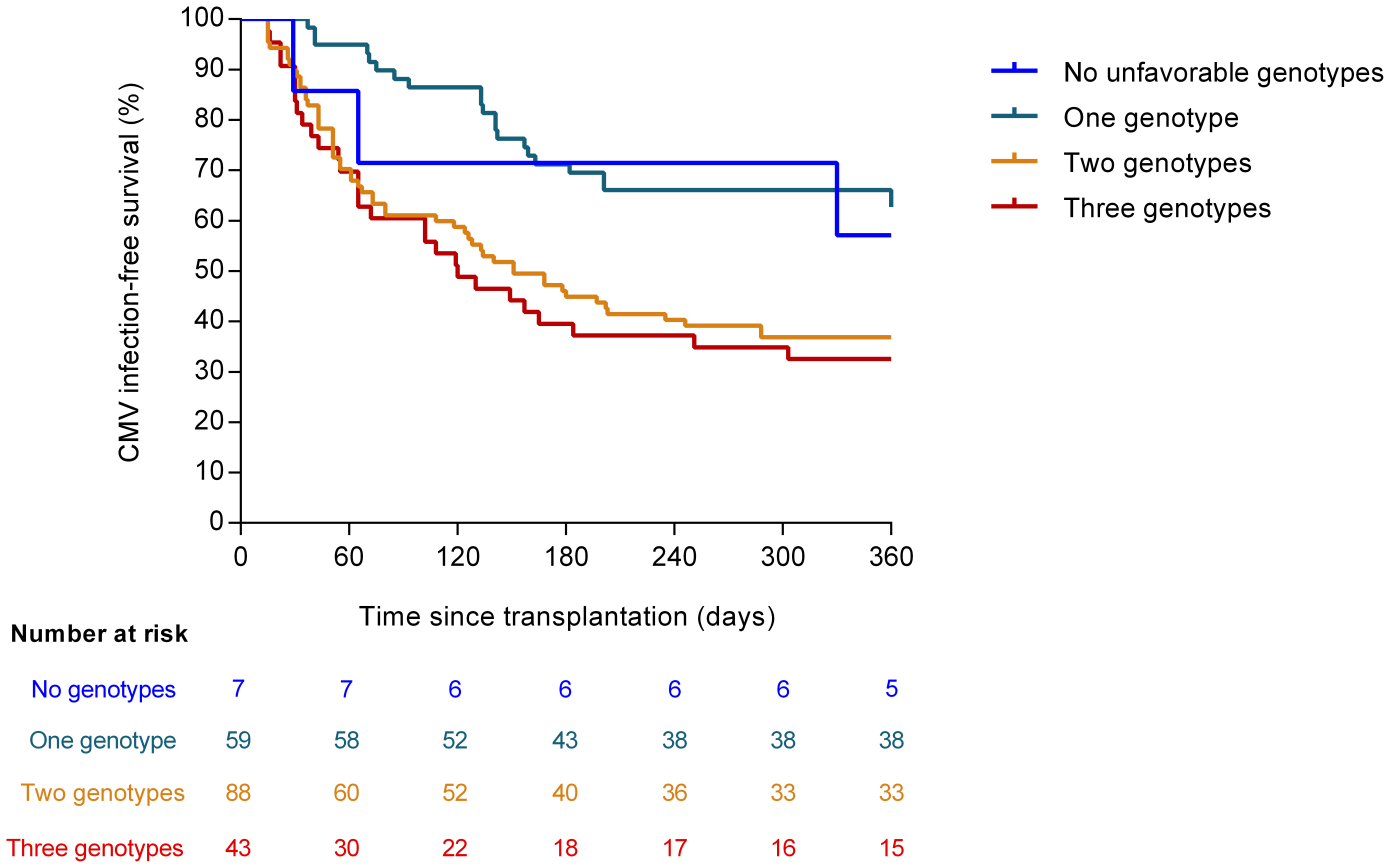
CMV infection-free survival according to the number of unfavorable genotypes in candidate SNPs (log-rank *P*-value = 0.002). The unfavorable genotypes were as follows: major C allele of *TLR3* (rs3775291) in homozygous or heterozygous state; minor G allele of *TLR9* (rs5743836) in homozygous or heterozygous state; and minor C allele of *TLR9* (rs352139) in homozygous or heterozygous state. CMV: cytomegalovirus.

We explored the additive impact on the risk of CMV infection of the number of risk genotypes in candidate SNPs. Genotypes were categorized as unfavorable as follows: the presence of the major C allele of *TLR3* (rs3775291) in either a homozygous or heterozygous state (concordant with a recessive protective effect for the minor T allele); the presence of the minor G allele of *TLR9* (rs5743836) in a homozygous or heterozygous state; and the presence of the minor C allele of *TLR9* (rs352139) also in a homozygous or heterozygous state (concordant in both latter cases with a dominant risk model). Patients were accordingly divided: no unfavorable genotypes (seven patients [3.6%]), one genotype (59 [29.9%]), two genotypes (88 [44.7%]) and three genotypes (43 [21.8%]). We observed that the CMV infection-free survival progressively decreased with the increasing number of unfavorable genotypes (one-year survival rates: 57.1% [no unfavorable genotypes], 62.7% [one genotype], 37.1% [two genotypes] and 32.5% [three genotypes]; log-rank test *P*-value = 0.002) (**Figure 2**). Similar results were observed for high-level CMV infection (**Figure S2**). After multivariable adjustment, the number of unfavorable genotypes in candidate SNPs remained significantly associated with the risk of CMV infection (aHR [per additional genotype]: 1.528; 95% CI: 1.212–1.927; *P*-value = 0.017).

### Effect of *TLR3* (rs3775291) and *TLR9* (rs5743836, rs352139) SNPs on the risk of CMV disease

We also explored the association between different allelic variants in candidate SNPs and CMV disease (secondary outcome). Concordant with the results obtained for overall and high-level CMV infection, carriers of the G allele of *TLR9* (rs5743836) in the homozygous or heterozygous state showed a trend toward a lower disease-free survival than homozygous carriers of the reference A allele (one-year survival rates: 83.2% versus 90.8%, respectively; log-rank test *P*-value = 0.149). The presence of the minor C allele of *TLR9* (rs352139) was also associated with a lower disease-free survival (one-year survival rates: 86.8% versus 93.2% for TC/CC and TT genotypes, respectively; log-rank test *P*-value = 0.194) (**Figure S3**). None of these differences, however, achieved statistical significance. The small number of events of CMV disease (n = 22) prevented us from performing multivariable adjustment.

### Correlation between CMV-CMI and candidate SNPs

**Figure 3:**
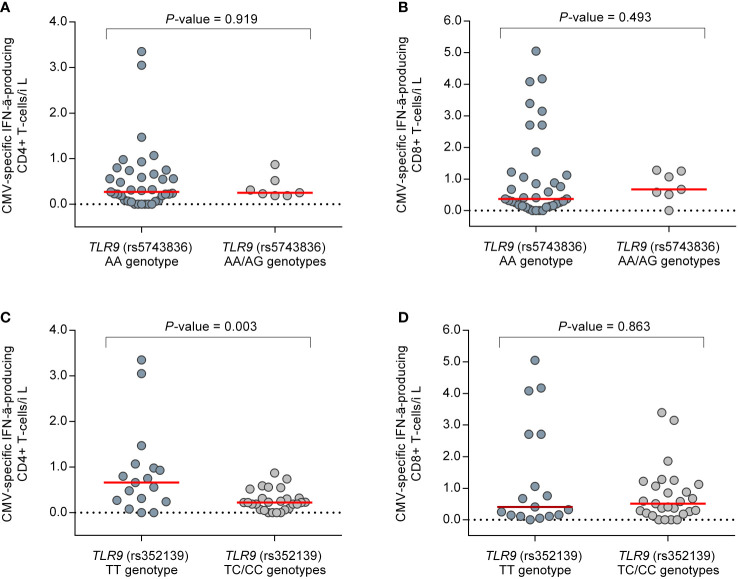
Comparison of CMV pp65 and IE-1-specific IFN-γ-producing CD4+ and CD8+ T-cell counts enumerated by intracellular cytokine stating in a subgroup of 31 patients (44 individual monitoring points) between selected genotypes of candidate SNPs: **(A)** CD4+ and **(B)** CD8+ T cells according to *TLR9* (rs5743836); **(C)** CD4+ and **(D)** CD8+ T cells according to *TLR9* (rs352139). Horizontal bars and whiskers represent median values and interquartile ranges, respectively. CMV, cytomegalovirus; IFN-γ, interferon-γ.

Finally, we investigated whether genetic polymorphisms in the *TLR3* and *TLR9* genes—which are essentially involved in activating the innate response—were correlated with the magnitude and functionality of CMV-CMI as the major effective components of the adaptive immunity against viruses. To this aim, we used two approaches: the ELISA-based QTF-CMV assay, which mainly detects CD8+ T-cell responses; and the reference ICS method (5, 28). In detail, the QTF-CMV assay was performed in 78 patients (39.6% of the overall cohort) at months 3, 4, and 5 (± one week), resulting in a total of 232 individual monitoring points. The enumeration of CMV pp65 and IE-1-specific IFN-γ-producing CD8+ and CD4+ T cells by ICS, on the other hand, was performed in a subgroup of 31 patients (15.7% of the cohort) at months 3 and 4, totaling 44 individual assessments. As depicted in **Figure S4**, there were no differences in the results of the QTF-CMV assay according to the presence of unfavorable genotypes in candidate SNPs, either by considering reactive responses (IFN-γ production [CMV minus nil] ≥0.2 IU/ml and ≥25% of nil) or by analyzing IFN-γ levels as a continuous variable. Additionally, no differences were observed in CMV-specific IFN-γ-producing CD8+ T-cell counts enumerated by ICS. Of note, carriers of the minor C allele of the *TLR9* (rs352139) SNP (TC/CC genotypes) had lower CMV-specific IFN-γ-producing CD4+ T-cell counts compared with those bearing the TT genotype (median of 0.2 [IQR: 0.1–0.3] versus 0.7 [IQR: 0.7–1.0] cells/μl, respectively; *P*-value = 0.003) (**Figure 3**). Comparison was not feasible for *TLR3* since only one patient with ICS data was homozygous for the minor T allele.

## Discussion

The contributing effect to the individual susceptibility to CMV of genetic variability in genes coding for immune mediators and receptors has been extended to the specific setting of SOT over the past years (10). This work contributes to this emerging field of research with two SNPs that have not been assessed in that population, such as *TLR3* (rs3775291) and *TLR9* (rs352139). Additionally, we have analyzed the *TLR9* (rs5743836) SNP in an attempt to validate the association with CMV infection previously reported in a multicenter cohort of seropositive KT recipients (20). Both *TLR3* and *TLR9* encode for endosomal PRRs that act cooperatively to orchestrate immune responses against viral pathogens, thus providing biological plausibility to our findings.

First, we have found that the presence in a homozygous state of the alternative allele of *TLR3* (rs3775291) (TT genotype) exerts a protective role on the risk of CMV viremia at any level and, with borderline significance, at high-level (≥1,000 IU/ml) infection. This polymorphism implies a C → T substitution which leads to the replacement of a conserved leucine with phenylalanine at position 412 (L412F). This missense mutation is mapped within the 14 leucine rich repeat domain of the TLR3 ectodomain and affects the binding capacity to dsRNA, resulting in a decreased signaling cascade compared to the wild-type form (21, 29). The *TLR3* (rs3775291) SNP has been linked with a protective effect against other viral infections. Kindberg et al. investigated the role of this polymorphism on the risk of tick-borne encephalitis (TBE), and reported that the functional form of TLR3—reflecting a homozygous state for the reference allele (CC genotype)—was overrepresented among patients with TBE virus infection as compared to healthy individuals. The authors suggested that homozygous carriers of the minor T allele would mount a reduced inflammatory response, attenuating the severity of TBE infection (30). Other authors found that the presence of the minor allele of *TLR3* (rs3775291) both in heterozygous or homozygous states conferred natural resistance against human immunodeficiency virus (HIV) infection in a cohort of 102 at-risk individuals. Interestingly, *in vitro* assays showed that HIV replication in peripheral mononuclear cells (PBMCs) carrying the L412F variant was significantly reduced compared with that of homozygous samples for the reference allele (31). The minor allelic variant at rs3775291 has been found to be more common among healthy HSV-2-seronegative individuals than in patients with genital HSV-2 infection, pointing to a protective phenotype, although no differences were observed in disease severity. Homozygous carriers of minor *TLR3* alleles expressed higher TLR3 mRNA levels in response to HSV-2 stimulation than those homozygous for the reference alleles, suggesting an impaired expression (32).

The inverse association observed in our cohort between the rs3775291 T allele in a recessive model and the occurrence of CMV events not only applied to the overall infection but was also shown for episodes of high-level viremia, although this protective effect could not be proven for CMV disease. Beyond the insufficient statistical power due to the small number of cases of disease, this lack of correlation may be explained by the fact that the allelic variation in *TLR3* acts at the innate level rather than shaping adaptive immunity, as supported by the observation that fibroblasts from TLR3-deficient patients show impaired IFN responses, although PBMCs from the same individuals adequately respond upon stimulation (32–34).

Taken together, the available evidence suggests that the protective role exerted by the *TLR3* (rs3775291) TT genotype would be due to a dampened inflammatory response mediated by the impaired TLR3 form, since it has been shown that the pro-inflammatory environment may act as a trigger for CMV reactivation (35, 36). Of note, the L412F substitution is only partially defective in terms of TLR function (21), which would be concordant with a recessive rather than dominant inheritance model.

Secondly, we have analyzed the *TLR9* (rs5743836) SNP investigated by our group in a previous multicenter cohort that included 315 CMV-seropositive KT recipients (20). In line with the results obtained from the OPERA Study, in actual experience we found that patients bearing the alternative G allele in a heterozygous or homozygous state (AG/GG genotypes) had a higher incidence of CMV infection compared to AA carriers, with this increased risk being even more evident for high-level viremia. This association, however, was not maintained after adjustment for clinical covariables (such as recipient and donor age, D+/R− serostatus, or use of valganciclovir prophylaxis). The *TLR9* (rs5743836) SNP—which is mapped in the promoter region of the gene affecting its transcription—has been related to increased susceptibility to other viral infections, such as dengue (37). Carvalho et al. reported that PBMCs heterozygous for the minor allelic variant of rs5743836 show a loop of TLR9/interleukin-6 signaling amplification upon CpG stimuli, resulting in increased B-cell activation and production of pro-inflammatory cytokines (22). It may be hypothesized that this milieu would in turn promote CMV reactivation.

Another relevant finding of our study is the risk conferred by the minor allelic variant of *TLR9* (rs352139) in the dominant model (TC/CC genotypes) for overall and high-level CMV infection and—without reaching statistical significance—CMV disease. This polymorphism (+1174 T>C) is located within intron 1 and has been associated with differences in *TLR9* expression at the transcriptional level (38). The combined presence of the minor SNP alleles at positions +1,174 and −1,486 can downregulate *TLR9* expression. Since rs352139 is an intronic polymorphism, it has been speculated that the C allele would generate an alternative splicing site that influences mRNA levels and therefore protein synthesis (39). Associations in the same direction between the *TLR9* (rs352139) SNP and infection have been reported, including maternal CMV infection in a cohort of Zimbabwean pregnant women in which homozygous carriers of the minor C allele were at higher risk than homozygous for the reference allele (40). Interestingly, the CT genotype of rs352139 has been recently shown to be associated with an increased risk of Epstein–Barr virus-related infectious mononucleosis, which points to a role in the susceptibility to herpesviruses different from CMV (41). Further studies on tuberculosis (42, 43) and malaria (44) have reached comparable conclusions.

Similar to TLR3, TLR9 is an endosomal PRR that is activated upon recognition of CpG motifs (12). This triggers the signaling cascade *via* the adaptor molecule MyD88 that stimulates the pro-inflammatory nuclear transcription factor NF-κβ and IFN production (12, 45). MyD88 is also an important mediator, inducing T-cell activation (46, 47). Taking this into consideration, it is plausible that polymorphisms in the *TLR9* gene lead to an impaired signaling cascade that would influence both innate and adaptive responses against CMV. This notion is supported by the novel observation that carriers of TC/CC genotypes of rs352139 exhibited lower counts of CMV-specific IFN-γ-producing CD4+ T cells by ICS than those bearing the TT genotype. The lack of differences in the QTF-CMV results between favorable and unfavorable genotypes may be explained by the fact that this assay essentially measures CD8+ T-cell responses (48). Indeed, CMV-specific CD8+ T-cell counts in the ICS assay did not differ either. These results would point to a specific impact of *TLR9* SNPs in CD4+ T-cell activation, which is consistent with the role shown for CD4+ T-cell-mediated responses in the long-term control with latent infection (49, 50).

Our study has some noteworthy limitations. The relatively small sample size has prevented us from performing a sensitivity analysis limited to patients not receiving antiviral prophylaxis. This limitation particularly applies to the analysis of CMV disease. Nevertheless, the type of prevention strategy was controlled for in the Cox models for overall and high-level CMV infection. Additionally, data on CMV-CMI by the QTF-CMV assay and ICS were retrospectively derived from previous studies (26, 27), which meant that this information was lacking in most genotyped patients. A significant HWE deviation was observed in the genotypic frequency of *TLR9* (rs5743836) SNP, which may have introduced some bias. Moreover, since the frequency of the minor allele G is very low in Caucasian ethnicities (0.14), only nine individuals in this cohort were carriers of the GG genotype. Both situations would have been overcome with a larger sample size. Finally, we did not genotype SNPs in the donors, although the clinical relevance is unclear due to the small amount of donor lymphoid tissue in the renal graft compared to other types of SOT such as the small bowel.

Nevertheless, our results are original since no evidence on the effect of *TLR3* (rs3775291) and *TLR9* (rs352139) SNPs on the risk of CMV events was available for the specific SOT population. Additionally, one of the main barriers to the implementation of genotyping into clinical practice is that most studies usually include a discovery cohort only and lack independent validation (10). In this line, we have reinforced our previous results concerning the significance of *TLR9* (rs5743836) on the occurrence of CMV viremia among KT recipients (20). Future studies should confirm the associations observed between these SNPs and the susceptibility of an individual to CMV infection and disease, as well as their potential implications for risk stratification and minimization. Finally, functional analysis should attempt to elucidate the biological mechanism underlying these associations.

## Data availability statement

The data that support the findings of this study are available upon reasonable request to the corresponding author.

## Ethics statement

The studies involving human participants were reviewed and approved by the Clinical Research Ethics Committee Hospital 12 de Octubre (Study protocol number 14/030). The patients/participants provided their written informed consent to participate in this study.

## Author contributions

NR and MF-R designed the study, performed statistical calculations, wrote the manuscript and supervised all aspects of the study. PP performed wet-lab analyses. TR-M collected the samples. IR-G, FL-M, EG, NP, AH, HT, RS, and AA involved in patients’ recruitment and data registration. IR-G, FL-M, RS, AA, and JA critically reviewed the manuscript and provided significant input and feedback on the draft manuscript. All authors listed have made a substantial, direct, and intellectual contribution to the work and approved it for publication.

## Funding

This work was supported by the Instituto de Salud Carlos III (ISCIII), Spanish Ministry of Science and Innovation (PIE13/00045, PI17/01120, PI20/01084)—co-financed by the European Development Regional Fund *“A way to achieve Europe”* and by the European Social Fund (ESF) *“The ESF invests in your future”*. IR-G. holds a research training contract “Río Hortega” (CM19/00163) and MF-R holds a research contract “Miguel Servet” (CP18/00073), both from the ISCIII, Spanish Ministry of Science and Innovation.

## Conflict of interest

The authors declare that the research was conducted in the absence of any commercial or financial relationships that could be construed as a potential conflict of interest.

## Publisher’s note

All claims expressed in this article are solely those of the authors and do not necessarily represent those of their affiliated organizations, or those of the publisher, the editors and the reviewers. Any product that may be evaluated in this article, or claim that may be made by its manufacturer, is not guaranteed or endorsed by the publisher.
