# Supplementary material for: Influence of single-nucleotide polymorphisms in TLR3 (rs3775291) and TLR9 (rs352139) on the risk of CMV infection in kidney transplant recipients
Source: Front Immunol. 2022 Jul 29;13:929995. doi: 10.3389/fimmu.2022.929995 (PMC9374175; doi:10.3389/fimmu.2022.929995)
Supplement: Supplementary file 1 [file DataSheet_1.docx]

***Supporting Material***

**Supplementary Methods**

*Immunosuppression and prophylaxis regimens*

Induction therapy with intravenous (IV) rabbit antithymocyte globulin (ATG-Fresenius®, 1.25 mg/Kg daily for 5-7 days) was used in the case of donation after circulatory death, with delayed initiation of tacrolimus on post-transplant day 6. Patients at high immunological risk also received ATG induction for 1-3 days with early tacrolimus initiation from day 0. Basiliximab induction (20 mg on days 0 and 4) with delayed tacrolimus introduction on day 5 was reserved to patients deemed at risk for nephrotoxicity (i.e. advanced age or comorbidities). The standard maintenance immunosuppression regimen consisted of tacrolimus (0.1 mg/Kg daily, adjusted to a target trough level of 10-15 ng/mL during the first month and 5-10 ng/mL thereafter), mycophenolic acid (360 mg twice daily), and prednisone (1 mg/Kg daily with progressive tapering). Conversion to mammalian target of rapamycin (typically everolimus) inhibitor with reduced-dose tacrolimus (target trough level of 3-6 ng/mL) was performed on an individual basis for recipients experiencing tacrolimus-related adverse effects, difficult-to-treat cytomegalovirus (CMV) of BK polyomavirus viremia, or malignancy.

All patients received a single IV dose of cefazolin (or ciprofloxacin in the case of hypersensitivity to ß-lactams) as preoperative antibiotic prophylaxis. Prophylaxis against *Pneumocystis jirovecii* pneumonia was based on trimethoprim-sulfamethoxazole (160/800 mg three times weekly) or monthly aerosolized pentamidine (300 mg) administered for 9 months [1]. Patients at high-risk for CMV disease received prophylaxis with oral valganciclovir (900 mg daily) for 3 months (R+ receiving induction therapy with ATG) or 6 months (serology mismatch [D+/R-]). Intermediate-risk patients (R+ without T-cell-depleting therapy) were monitored every 2-4 weeks for CMV viremia with a PCR-based assay and preemptive therapy with intravenous ganciclovir (5 mg/Kg/12 hours) or valganciclovir (900 mg/12 hours) was given for at least 2 weeks in presence of high-level (>1,000 IU/mL) or increasing viral loads [2].

*Supplementary study definitions*

The diagnosis of CMV infection required polymerase chain reaction (PCR)-confirmed CMV replication regardless of the presence of attributable symptoms. The definition of CMV disease comprised both viral syndrome and end-organ disease. Viral syndrome was defined by the presence of CMV infection plus fever plus at least one of the following: leukopenia (white blood cell [WBC] count <3.50 x 10^3^ cells/μL if baseline WBC count was ≥4.00 x 10^3^ cells/μL or a decrease >20% if baseline WBC count was <4.00 x 10^3^ cells/μL); atypical lymphocytosis (≥5%); thrombocytopenia (platelet count <100 x 10^3^ cells/μL if baseline count was ≥115 x 10^3^ cells/μL or a decrease >20% if baseline platelet count was <115 x 10^3^ cells/μL); or elevation of ALT or AST of more than 2 times the upper limit of normal. End-organ disease included probable or proven categories (e.g. colitis, hepatitis, pneumonitis, gastritis or retinitis), with the latter requiring the documentation of CMV replication in tissue specimens by viral culture, immunohistochemistry, histopathology or DNA *in situ* hybridization, in the presence of attributable clinical manifestations [3]. Graft function was assessed by estimated glomerular filtration rate using the abbreviated Modification of Diet in Renal Disease (MDRD-4) equation [4]. Delayed graft function was defined as the need for dialysis within the first post-transplant week. Acute graft rejection was diagnosed by histological examination if possible or by response to empirical antirejection treatment.

*Assessment of CMV viral load*

In brief, 200 μL of whole blood were used for DNA extraction using a NucliSENS® easyMag® instrument (bioMérieux Diagnostics, Marcy l’Etoile, France), according to the manufacturer's instructions. A commercial real-time PCR assay (RealStar® CMV PCR kit 1.0, Altona Diagnostics GmbH, Hamburg, Germany) was used for viral DNA quantification.

*Assessment of CMV-specific T-cell-mediated immunity by the QuantiFERON®-CMV assay*

The CMV-CMI was first investigated with the commercial QuantiFERON®-CMV (QTF-CMV) assay (Qiagen GmbH, Hilden, Germany), as described elsewhere [5]. Briefly, one-mL aliquots of heparinized whole blood were collected into three QTF-CMV collection tubes: one of them contained a pool of 22 CMV peptides mapped within pp65, pp50, immediate-early (IE)-1, IE-2 and glycoprotein B antigens and restricted by several widespread HLA class I molecules (“CMV tube”); a second tube contained phytohemagglutinin (PHA) as a nonspecific lymphocyte mitogen (“mitogen tube” [positive control]); and the third tube contained heparin only (“nil tube” [negative control]). These tubes were shaken vigorously for 5 seconds and then incubated for 18 to 24 hours at 37°C. Following incubation, supernatants were harvested at 3000 rpm for 15 minutes and frozen at -80°C until analysis. Interferon (IFN)-γ levels were measured by an enzyme-linked immunosorbent assay. A standard curve was generated for each run. The results of the assay were interpreted according to the criteria established by the manufacturer: (i) nonreactive at <0.2 IU/mL (CMV minus nil) or ≥0.2 IU/mL and <25% of nil tube plus ≥0.5 IU/mL (mitogen minus nil), (ii) reactive at ≥0.2 IU/mL (CMV minus nil) and ≥25% of nil, and (iii) indeterminate at <0.2 IU/mL (CMV minus nil) or ≥0.2 IU/mL and <25% of nil tube plus <0.5 IU/mL (mitogen minus nil).

*Assessment of CMV-specific T-cell-mediated immunity by intracellular cytokine staining*

Whole blood specimens were collected in sodium heparin tubes and processed within 24 hours. CMV-specific IFN-γ-producing CD8+ and CD4+ T-cells was carried out using the BD FastImmune^TM^ CD4 and CD8 Intracellular Cytokine Detection kit (Cat no: 337185 and 346049, respectively; BD Biosciences, San Jose, CA) following manufacturer´s instructions. A volume of 0.5 mL of whole blood was simultaneously stimulated for 6 hours with 2 sets of 15-mer overlapping peptides (with an 11-aa overlap) encompassing the entire sequence of pp65 and IE-1 peptides (138 and 120 peptides, respectively [with 2 μg/mL per peptide]), both from JPT Peptide Technologies GmbH (Berlin, Germany) in the presence of costimulatory monoclonal antibodies targeting CD28 and CD49d. Samples mock stimulated with phosphate-buffered saline (PBS)/dimethyl sulfoxide solution (without peptides) and costimulatory antibodies or stimulated with PHA (1 mg/mL) (Sigma-Aldrich, St. Louis, MO) were run in parallel in all experiments. Brefeldin A (5 μg/mL) was added for the last 4 hours of incubation. Stimulated specimens were fixed with BD FACS^TM^ Lysing Solution (BD Biosciences) and frozen at -80 ºC. All analyses were done in batches within one month after stimulation. To this end, specimens were thawed at 37 ºC, washed in PBS-2% fetal calf serum, permeabilized (BD FACS^TM^ permeabilizing solution 2) and stained with a combination of labeled monoclonal antibodies (anti-CD3-APC-Cy7, anti-CD69-PE, anti-CD4 or CD8-PerCP-Cy5.5, and anti-IFN-γ-FITC) for 30 minutes at room temperature. All monoclonal antibodies and solutions were purchased from BD Biosciences (Cat no: 561800, 557050, 560650, 560662 and 552882, respectively). Appropriate isotype controls were used (BD FastImmune γ2a/γ1/CD8/CD3). Cells were then washed again, resuspended in 200 μL of 1% paraformaldehyde in PBS, and analyzed within 2 hours on a FACSCalibur flow cytometer using CellQuest software (BD Biosciences Immunocytometry Systems). Cells were first gated for lymphocytes (SSC-A versus FSC-A). The lymphocyte population was analyzed for their surface expression of CD3, CD4 and CD8. CD3+CD8+ or CD3+CD46 cells were further gated for their expression of the activation marker CD69 and intracellular IFN-γ. The total number of each T-cell subpopulation was calculated by multiplying the corresponding percentage of CMV-specific cells (after background subtraction) by the absolute number of CD8+ and CD4+ T-cells [6].

*References:*

1. Fishman JA, Gans H; AST Infectious Diseases Community of Practice. *Pneumocystis jiroveci* in solid organ transplantation: Guidelines from the American Society of Transplantation Infectious Diseases Community of Practice. *Clin Transplant.* 2019;33(9):e13587.
2. Torre-Cisneros J, Aguado JM, Castón JJ, Almenar L, Alonso A, Cantisán S, et al. Management of cytomegalovirus infection in solid organ transplant recipients: SET/GESITRA-SEIMC/REIPI recommendations. *Transplant Rev (Orlando).* 2016;30(3):119-43.
3. Ljungman P, Boeckh M, Hirsch HH, Josephson F, Lundgren J, Nichols G, et al. Definitions of cytomegalovirus infection and disease in transplant patients for use in clinical trials. *Clin Infect Dis.* 2017;64:87-91.
4. Levey AS, Bosch JP, Lewis JB, Greene T, Rogers N, Roth D. A more accurate method to estimate glomerular filtration rate from serum creatinine: a new prediction equation. Modification of Diet in Renal Disease Study Group. *Ann Intern Med.* 1999;130:461-70.
5. Fernández-Ruiz M, Rodríguez-Goncer I, Parra P, Ruiz-Merlo T, Corbella L, López-Medrano F, et al. Monitoring of CMV-specific cell-mediated immunity with a commercial ELISA-based interferon-gamma release assay in kidney transplant recipients treated with antithymocyte globulin. *Am J Transplant.* 2020;20:2070-80.
6. Fernández-Ruiz M, Giménez E, Vinuesa V, Ruiz-Merlo T, Parra P, Amat P, et al. Regular monitoring of cytomegalovirus-specific cell-mediated immunity in intermediate-risk kidney transplant recipients: predictive value of the immediate post-transplant assessment. *Clin Microbiol Infect*. 2019;25:381 e1-e10.

**Supplementary Results**

**Table S1**. Genotypic frequencies of the explored SNPs.

| **Gene (SNP database ID number)** | **Genotype** | **Genotypic frequencies** (n [%]) | | | **X^2^** | ***P*-value** |
| --- | --- | --- | --- | --- | --- | --- |
|  |  | Expected according to the HWE | Observed | |  |  |
| *TLR3* (rs3775291) | CC | 92.5 | | 98 |  |  |
|  | CT | 85.0 | | 74 | 3.29 | >0.05 |
|  | TT | 19.5 | | 25 |  |  |
| *TLR9* (rs5743836) | AA | 139.0 | | 143 |  |  |
|  | AG | 52.9 | | 45 | 4.42 | <0.05 |
|  | GG | 5.0 | | 9 |  |  |
| *TLR9* (rs352139) | TT | 53.3 | | 59 |  |  |
|  | TC | 98.3 | | 87 | 2.62 | >0.05 |
|  | CC | 45.3 | | 51 |  |  |
| HWE: Hardy-Weinberg equilibrium; ID: identification; SNP: single-nucleotide polymorphism; TLR: toll-like receptor. | | | | | | |

**Table S2**. One-year cumulative incidence of high-level CMV infection (≥1,000 IU/mL) according to recessive and dominant models for the minor alleles of candidate SNPs.

| **Gene (SNP database ID number)** | Model | Genotype | **High-level CMV infection by month 12** (n [%]) | | ***P*-value** |
| --- | --- | --- | --- | --- | --- |
|  |  |  | No infection  (n = 125) | Infection  (n = 72) |  |
| *TLR3* (rs3775291) | Dominant | CC | 61 (48.8) | 37 (51.4) | 0.726 |
|  |  | CT/TT | 64 (51.2) | 35 (48.6) |  |
|  | Recessive | CC/CT | 105 (84.0) | 67 (93.1) | 0.066 |
|  |  | TT | 20 (16.0) | 5 (6.9) |  |
| *TLR9* (rs5743836) | Dominant | AA | 97 (77.6) | 46 (63.9) | 0.038 |
|  |  | AG/GG | 28 (22.4) | 26 (36.1) |  |
|  | Recessive | AA/AG | 121 (96.8) | 67 (93.1) | 0.291 |
|  |  | GG | 4 (3.2) | 5 (6.9) |  |
| *TLR9* (rs352139) | Dominant | TT | 44 (35.2) | 15 (20.8) | 0.034 |
|  |  | TC/CC | 81 (64.8) | 57 (79.2) |  |
|  | Recessive | TT/TC | 98 (78.4) | 48 (66.7) | 0.070 |
|  |  | CC | 27 (21.6) | 24 (33.3) |  |
| CMV: cytomegalovirus; ID: identification; SNP: single-nucleotide polymorphism; TLR: toll-like receptor. | | | | | |

**Table S3**. Univariate analysis of factors predicting the occurrence of CMV infection during the first post-transplant year.

|  | No CMV infection  (n = 88) | CMV infection  (n = 109) | *P*-value | Univariate analysis | | |
| --- | --- | --- | --- | --- | --- | --- |
|  |  |  |  | HR | 95% CI | *P*-value |
| Age of recipient, years [mean ± SD] | 49.7 ± 16.6 | 59.4 ± 12.9 | <0.0001 | 1.396^c^ | 1.224 – 1.592 | <0.0001 |
| Gender (male) [n (%)] | 62 (70.5) | 79 (72.5) | 0.754 |  |  |  |
| BMI of recipient, Kg/m^2^ [mean ± SD]^a^ | 24.8 ± 4.3 | 27.0 ± 12.4 | 0.127 |  |  |  |
| Pre-transplant diabetes mellitus [n (%)] | 24 (27.3) | 33 (30.3) | 0.644 |  |  |  |
| Pre-transplant coronary heart disease [n (%)] | 11 (12.5) | 10 (9.2) | 0.452 |  |  |  |
| Pre-transplant chronic lung disease [n (%)] | 10 (11.4) | 17 (15.6) | 0.390 |  |  |  |
| Previous kidney transplantation [n (%)] | 14 (15.9) | 12 (11.0) | 0.312 |  |  |  |
| Pre-transplant renal replacement therapy [n (%)] | 78 (88.6) | 97 (89.0) | 0.937 |  |  |  |
| Time on dialysis, months [median (IQR)] | 18.9 (8.6 – 47.4) | 14.2 (9.2 – 32.6) | 0.349 |  |  |  |
| Positive HCV serostatus [n (%)] | 7 (8.2) | 8 (7.4) | 0.831 |  |  |  |
| Age of donor, years [mean ± SD] | 49.5 ± 15.3 | 57.4 ± 14.8 | <0.0001 | 1.309^c^ | 1.149 – 1.492 | <0.0001 |
| DCD donor [n (%)] | 25 (28.4) | 20 (18.3) | 0.094 |  |  |  |
| Living donor [n (%)] | 16 (18.2) | 10 (9.2) | 0.063 |  |  |  |
| CMV serostatus D+/R- [n (%)] | 16 (18.8) | 7 (6.5) | 0.009 | 0.423 | 0.196 – 0.910 | 0.028 |
| CMV serostatus D+/R+ [n (%)] | 57 (67.1) | 91 (84.3) | 0.005 | 2.186 | 1.300 – 3.674 | 0.003 |
| CMV serostatus D-/R+ [n (%)] | 12 (14.1) | 10 (9.3) | 0.292 |  |  |  |
| Cold ischemia time, hours [mean ± SD]^b^ | 17.4 ± 7.5 | 15.5 ± 8.5 | 0.096 |  |  |  |
| Number of HLA mismatches [median (IQR)] | 4 (3 – 5) | 5 (3 – 5) | 0.254 |  |  |  |
| Antiviral prophylaxis [n (%)] | 61 (69.3) | 50 (45.9) | 0.001 | 0.367 | 0.250 – 0.537 | <0.0001 |
| Induction therapy with ATG [n (%)] | 50 (56.8) | 42 (38.5) | 0.011 | 0.468 | 0.318 – 0.690 | <0.0001 |
| Delayed graft function [n (%)]^c^ | 42 (47.7) | 56 (51.9) | 0.566 |  |  |  |
| eGFR at month 1, mL/min/1.73 m^2^ [mean ± SD] | 45.9 ± 19.9 | 35.6 ± 16.6 | <0.0001 | 0.980^d^ | 0.970 – 0.991 | <0.0001 |
| Use of mTOR inhibitor [n (%)] |  |  |  |  |  |  |
| During the first 6 post-transplant months | 2 (2.3) | 4 (3.7) | 0.579 |  |  |  |
| During the first post-transplant year | 4 (4.5) | 12 (11.0) | 0.099 |  |  |  |
| Acute graft rejection [n (%)] |  |  |  |  |  |  |
| During the first post-transplant month | 2 (2.3) | 5 (4.6) | 0.464 |  |  |  |
| During the first 3 post-transplant months | 4 (4.5) | 6 (5.5) | 0.760 |  |  |  |
| During the first 6 post-transplant months | 6 (6.8) | 12 (11.0) | 0.310 |  |  |  |
| ATG: antithymocyte globulin; BMI: body mass index; CI: confidence interval; CMV: cytomegalovirus; D: donor; DCD: donation after circulatory death; eGFR: estimated glomerular filtration rate; HLA: human leukocyte antigen; HR: hazard ratio; IQR: interquartile range; mTOR: mammalian target of rapamycin; R: recipient; SD: standard deviation.  ^a^ Data on BMI was not available for 17 patients.  ^b^ Data on cold ischemia time was not available for 5 patients.  ^c^ HR per each ten-year increment.  ^d^ HR per each one-mL/min/1.73 m^2^ increment. | | | | | | |

**Table S4**. Univariate analysis of factors predicting the occurrence of high-level CMV infection (≥1,000 IU/mL) during the first post-transplant year.

|  | No high-level CMV infection  (n = 121) | High-level CMV infection  (n = 76) | *P*-value | Univariate analysis | | |
| --- | --- | --- | --- | --- | --- | --- |
|  |  |  |  | HR | 95% CI | *P*-value |
| Age of recipient, years [mean ± SD] | 52.4 ± 16.1 | 59.3 ± 13.2 | 0.001 | 1.346^c^ | 1.145 – 1.582 | <0.0001 |
| Gender (male) [n (%)] | 84 (69.4) | 57 (75.0) | 0.398 |  |  |  |
| BMI of recipient, Kg/m^2^ [mean ± SD]^a^ | 25.3 ± 4.0 | 27.2 ± 14.8 | 0.196 |  |  |  |
| Pre-transplant diabetes mellitus [n (%)] | 33 (27.3) | 24 (31.6) | 0.516 |  |  |  |
| Pre-transplant coronary heart disease [n (%)] | 13 (10.7) | 8 (10.5) | 0.876 |  |  |  |
| Pre-transplant chronic lung disease [n (%)] | 13 (10.7) | 14 (18.4) | 0.127 |  |  |  |
| Previous kidney transplantation [n (%)] | 19 (15.7) | 7 (9.2) | 0.274 |  |  |  |
| Pre-transplant renal replacement therapy [n (%)] | 108 (89.3) | 67 (88.2) | 0.812 |  |  |  |
| Time on dialysis, months [median (IQR)] | 18.8 (8.9 – 44.0) | 12.8 (8.9 – 32.6) | 0.403 |  |  |  |
| Positive HCV serostatus [n (%)] | 10 (8.5) | 5 (6.7) | 0.647 |  |  |  |
| Age of donor, years [mean ± SD] | 51.5 ± 15.7 | 57.7 ± 14.3 | 0.006 | 1.265^c^ | 1.080 – 1.482 | 0.004 |
| DCD donor [n (%)] | 31 (25.6) | 14 (18.4) | 0.241 |  |  |  |
| Living donor [n (%)] | 19 (15.7) | 7 (9.2) | 0.190 |  |  |  |
| CMV serostatus D+/R- [n (%)] | 17 (14.4) | 6 (8) | 0.181 |  |  |  |
| CMV serostatus D+/R+ [n (%)] | 86 (72.9) | 62 (82.7) | 0.117 |  |  |  |
| CMV serostatus D-/R+ [n (%)] | 15 (12.7) | 7 (9.3) | 0.472 |  |  |  |
| Cold ischemia time, hours [mean ± SD]^b^ | 16.4 ± 8.2 | 16.9 ± 7.6 | 0.668 |  |  |  |
| Number of HLA mismatches [median (IQR)] | 4 (3-5) | 5 (3-5) | 0.371 |  |  |  |
| Antiviral prophylaxis [n (%)] | 78 (64.5) | 33 (43.4) | 0.004 | 0.368 | 0.229 – 0.590 | <0.0001 |
| Induction therapy with ATG [n (%)] | 64 (52.9) | 28 (36.8) | 0.028 | 0.464 | 0.285 – 0.754 | 0.002 |
| Delayed graft function [n (%)] | 57 (47.1) | 41 (54.7) | 0.304 |  |  |  |
| eGFR at month 1, mL/min/1.73 m^2^ [mean ± SD] | 44.2 ± 19.8 | 33.9 ± 15.1 | <0.001 | 0.974^d^ | 0.961 – 0.988 | <0.001 |
| Use of mTOR inhibitor [n (%)] |  |  |  |  |  |  |
| During the first 6 post-transplant months | 2 (1.7) | 4 (5.3) | 0.151 |  |  |  |
| During the first post-transplant year | 4 (3.3) | 12 (15.8) | 0.002 | 3.170 | 1.704 – 5.895 | <0.001 |
| Acute graft rejection [n (%)] |  |  |  |  |  |  |
| During the first post-transplant month | 4 (3.3) | 3 (3.9) | 0.813 |  |  |  |
| During the first 3 post-transplant months | 6 (5.0) | 4 (5.3) | 0.924 |  |  |  |
| During the first 6 post-transplant months | 9 (7.4) | 9 (11.8) | 0.296 |  |  |  |
| ATG: antithymocyte globulin; BMI: body mass index; CI: confidence interval; CMV: cytomegalovirus; D: donor; DCD: donation after circulatory death; eGFR: estimated glomerular filtration rate; HLA: human leukocyte antigen; HR: hazard ratio; IQR: interquartile range; mTOR: mammalian target of rapamycin; R: recipient; SD: standard deviation.  ^a^ Data on BMI was not available for 17 patients.  ^b^ Data on cold ischemia time was not available for 5 patients.  ^c^ HR per each ten-year increment.  ^d^ HR per each one-mL/min/1.73 m^2^ increment. | | | | | | |

**Table S5.** Multivariable Cox regression models assessing the impact of selected SNPs on the incidence of high-level CMV infection (≥1,000 IU/mL) during the first post-transplant year.

| **Genotype** | aHR^a^ | 95% CI | *P-*value |
| --- | --- | --- | --- |
| TT genotype of *TLR3* (rs3775291) SNP (vs. CC/CT) | 0.409 | 0.162 – 1.036 | 0.059 |
| AG/GG genotype of *TLR9* (rs5743836) SNP (vs. AA) | 1.359 | 0.826 – 2.235 | 0.227 |
| TC/CC genotype of *TLR9* (rs352139) SNP (vs. CC) | 1.859 | 1.039 – 3.328 | 0.037 |
| aHR: adjusted hazard ratio; CI: confidence interval; SNP: single-nucleotide polymorphism; TLR: toll-like receptor.  ^a^ Model adjusted for recipient and donor age, receipt of valganciclovir prophylaxis, graft function at month 1, reintervention within the first month and mTOR inhibitor conversion during the first post-transplant year. Induction therapy with ATG was not entered into the model due to its high collinearity with the use of antiviral prophylaxis. | | | |


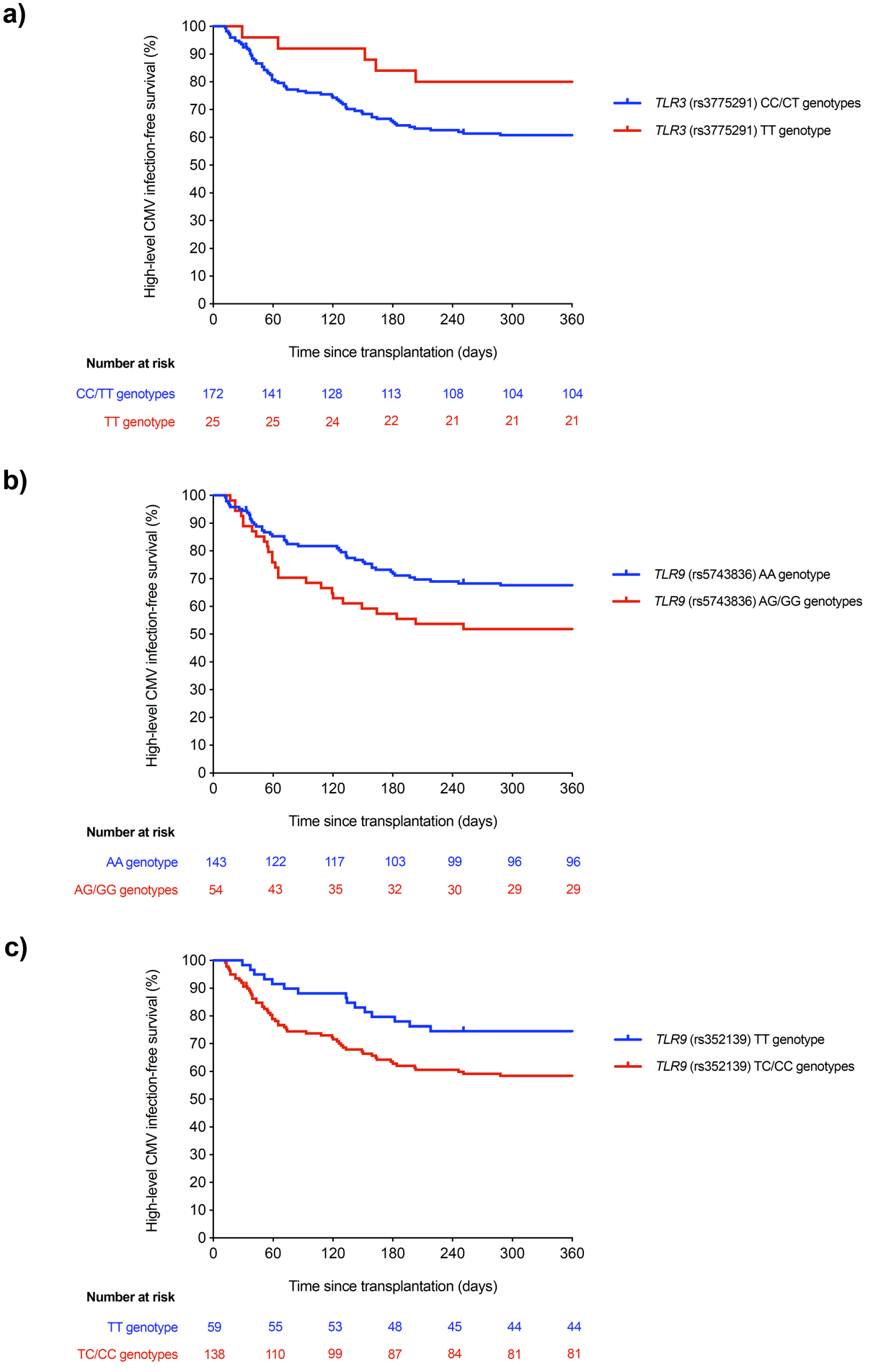
**Figure S1**. Comparison of high-level CMV infection (≥1,000 IU/mL)-free survival according to selected genotypes of candidate SNPs: **a)** *TLR3* (rs3775291) (log-rank test *P*-value = 0.066), **b)** *TLR9* (rs5743836) (log-rank test *P*-value = 0.031), and **c)** *TLR9* (rs352139) (log-rank test *P*-value = 0.024). CMV: cytomegalovirus; SNP: single-nucleotide polymorphism.


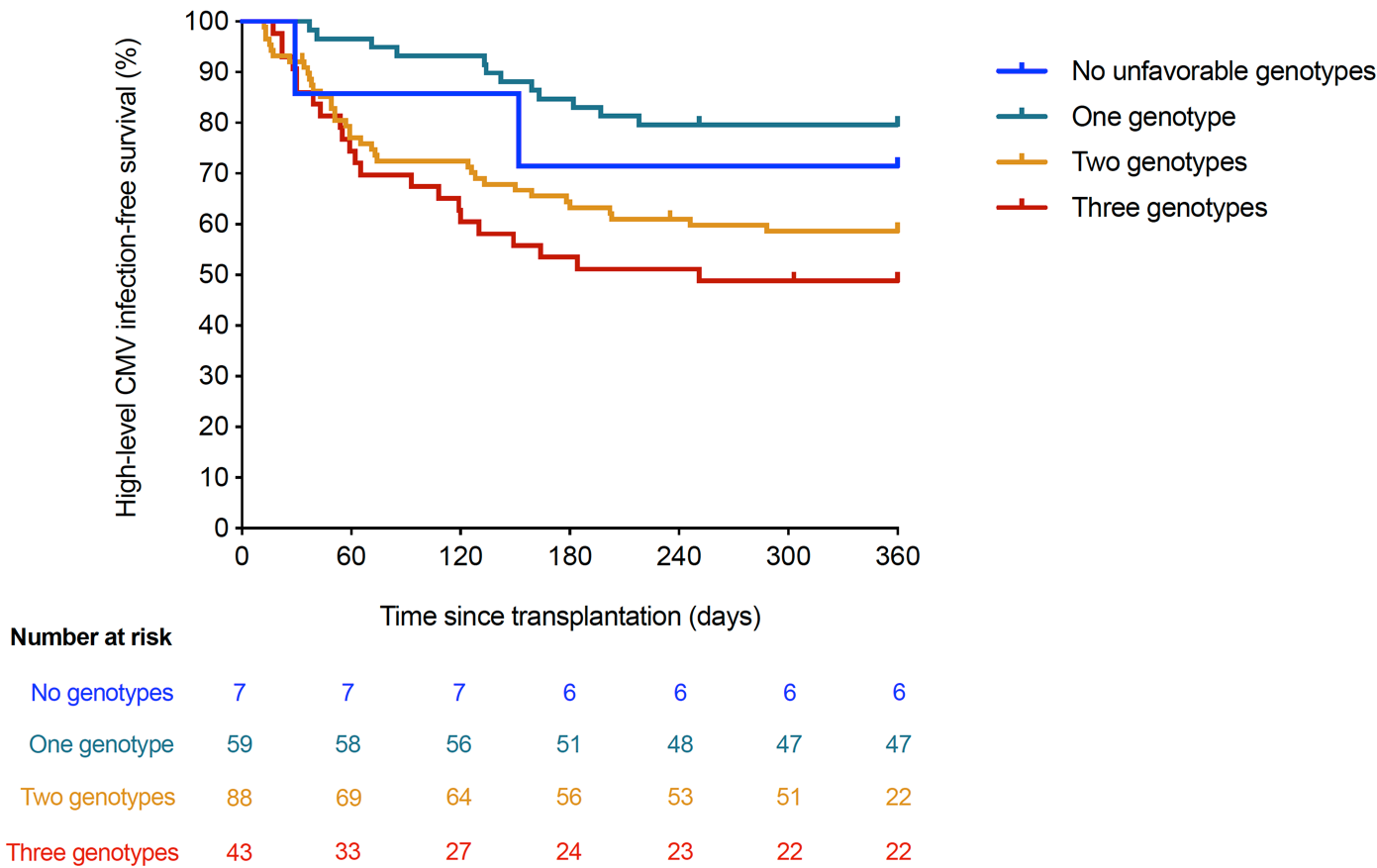
**Figure S2**. High-level CMV infection-free survival according to the number of unfavorable genotypes in candidate SNPs (log-rank *P*-value = 0.005). The unfavorable genotypes were as follows: major C allele of *TLR3* rs3775291 in homozygous or heterozygous state; minor G allele of *TLR9* rs5743836 in homozygous or heterozygous state; and minor C allele of *TLR9* (rs352139) in homozygous or heterozygous state. CMV: cytomegalovirus.


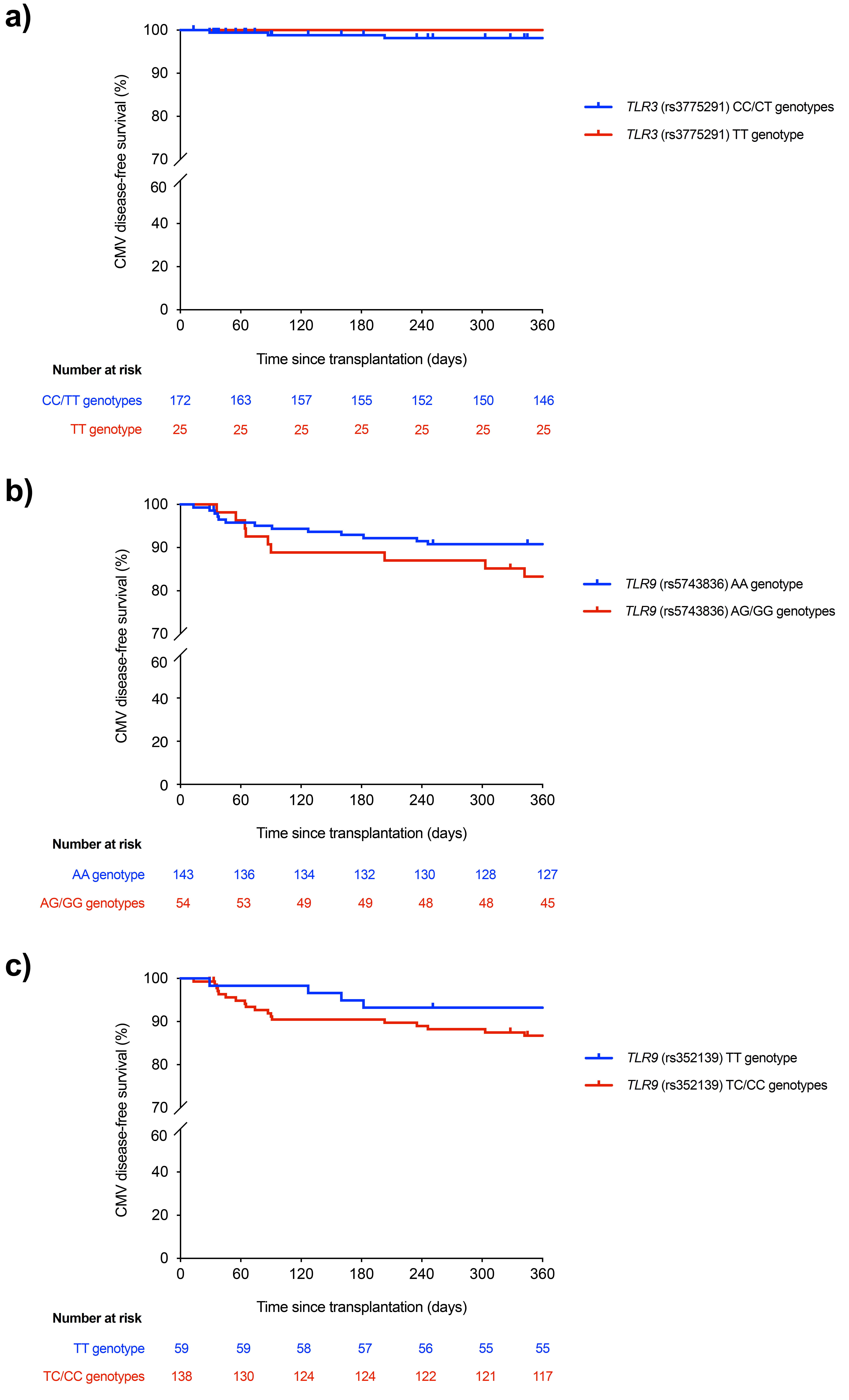
**Figure S3.** Comparison of CMV disease-free survival according to selected genotypes of candidate SNPs: **a)** *TLR3* (rs3775291) (log-rank test *P*-value = 0.896), **b)** *TLR9* (rs5743836) (log-rank test *P*-value = 0.149), and **c)** *TLR9* (rs352139) (log-rank test *P*-value = 0.194).

**
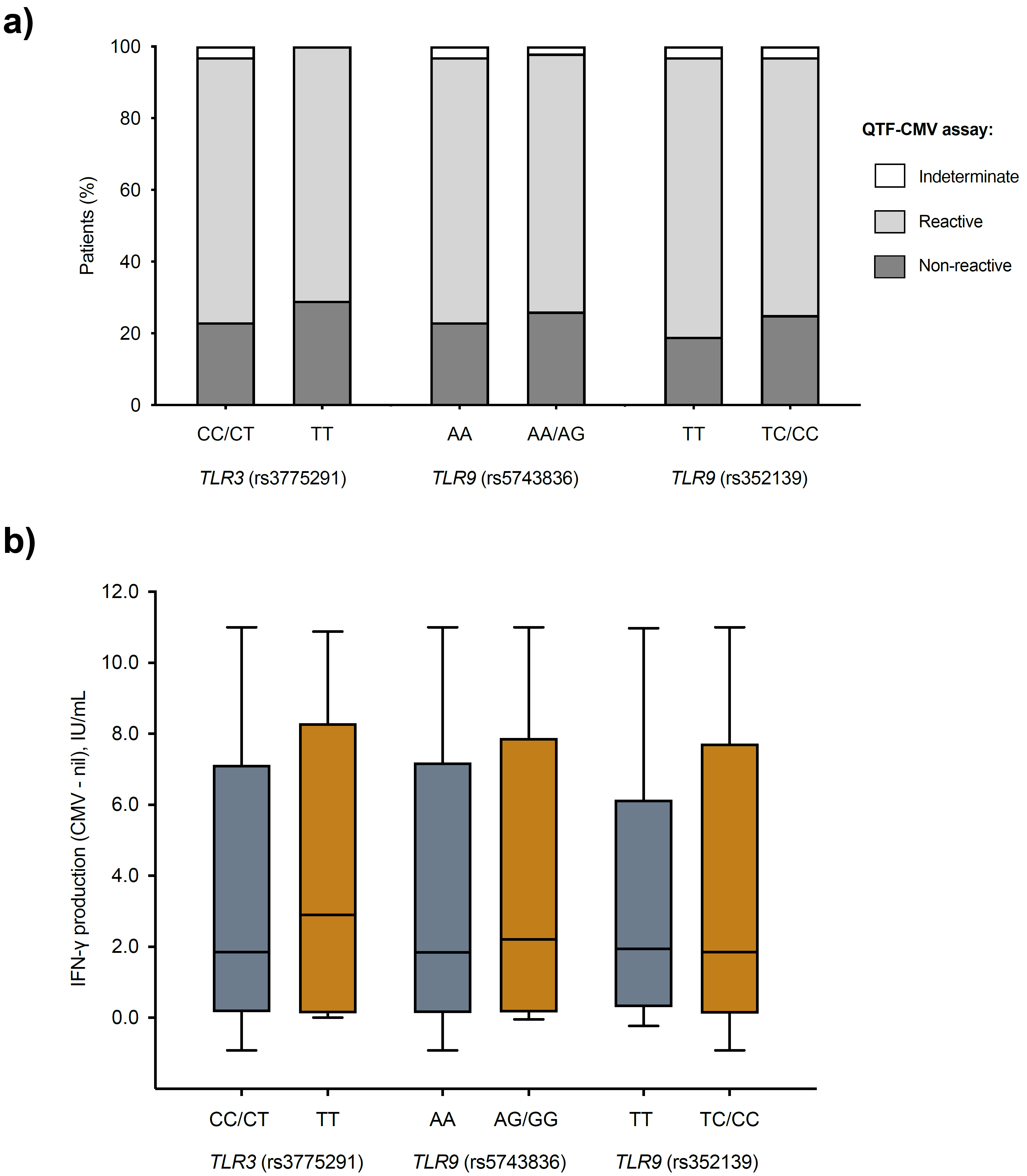
Figure S4.** Comparison of the results of the commercial ELISA-based QuantiFERON®-CMV assay in a subgroup of 78 patients (232 individual monitoring points) between selected genotypes of candidate SNPs: **a)** assay reactivity as per the interpretative criteria proposed by the manufacturer; **b)** interferon-γ production (CMV – nil tube) as a continuous variable; horizontal lines within the boxes represent the median, outer horizontal lines of the boxes are the 25^th^ and 75^th^ quartiles, and horizontal lines of the whiskers are the Tukey inner fences. None of the comparisons achieved statistical significance. QTF-CMV: QuantiFERON®-CMV assay; IFN-γ: interferon-γ.
